# A Review of Data Mining Strategies by Data Type, with a Focus on Construction Processes and Health and Safety Management

**DOI:** 10.3390/ijerph21070831

**Published:** 2024-06-26

**Authors:** Antonella Pireddu, Angelico Bedini, Mara Lombardi, Angelo L. C. Ciribini, Davide Berardi

**Affiliations:** 1 Department of Technological Innovations and Safety of Plants, Products and Anthropic Settlements (DIT), Italian National Institute for Insurance against Accidents at Work, Inail, 00144 Rome, Italy; 2 Department of Chemical Engineering Materials Environment (DICMA), Sapienza-University of Rome, 00184 Rome, Italy; mara.lombardi@uniroma1.it (M.L.); davide.berardi@uniroma1.it (D.B.); 3 Department of Civil Engineering, Architecture, Land, Environment and Mathematics (DICATAM), Brescia University, 25121 Brescia, Italy; angelo.ciribini@unibs.it

**Keywords:** clustering, principal component analysis (PCA), meta-analysis, construction industry, data mining, machine learning, prediction models, workplace safety, smart technology (ST), state of the art.

## Abstract

Increasingly, information technology facilitates the storage and management of data useful for risk analysis and event prediction. Studies on data extraction related to occupational health and safety are increasingly available; however, due to its variability, the construction sector warrants special attention. This review is conducted under the research programs of the National Institute for Occupational Accident Insurance (Inail). Objectives: The research question focuses on identifying which data mining (DM) methods, among supervised, unsupervised, and others, are most appropriate for certain investigation objectives, types, and sources of data, as defined by the authors. Methods: Scopus and ProQuest were the main sources from which we extracted studies in the field of construction, published between 2014 and 2023. The eligibility criteria applied in the selection of studies were based on the Preferred Reporting Items for Systematic Review and Meta-Analyses (PRISMA). For exploratory purposes, we applied hierarchical clustering, while for in-depth analysis, we used principal component analysis (PCA) and meta-analysis. Results: The search strategy based on the PRISMA eligibility criteria provided us with 63 out of 2234 potential articles, 206 observations, 89 methodologies, 4 survey purposes, 3 data sources, 7 data types, and 3 resource types. Cluster analysis and PCA organized the information included in the paper dataset into two dimensions and labels: “supervised methods, institutional dataset, and predictive and classificatory purposes” (correlation 0.97–8.18 × 10^−1^; *p*-value 7.67 × 10^−55^–1.28 × 10^−22^) and the second, Dim2 “not-supervised methods; project, simulation, literature, text data; monitoring, decision-making processes; machinery and environment” (corr. 0.84–0.47; *p*-value 5.79 × 10^−25^–-3.59 × 10^−6^). We answered the research question regarding which method, among supervised, unsupervised, or other, is most suitable for application to data in the construction industry. Conclusions: The meta-analysis provided an overall estimate of the better effectiveness of supervised methods (Odds Ratio = 0.71, Confidence Interval 0.53–0.96) compared to not-supervised methods.

## 1. Introduction

The activities attributable to the construction sector, according to the International Labour Organisation (ILO) classification, are as follows: (i) building, including excavation and the construction, structural alteration, renovation, repair, maintenance (including cleaning and painting), and demolition of all types of buildings or structures; (ii) civil engineering, including excavation and the construction, structural alteration, repair, maintenance, and demolition of structures such as airports, docks, harbors, inland waterways, dams, river, avalanche, and sea defense works, roads and highways, railways, bridges, tunnels, viaducts, and works related to the provision of services such as communications, drainage, sewerage, water, and energy supplies; and (iii) the erection and dismantling of prefabricated buildings and structures, as well as the manufacturing of prefabricated elements on construction sites [1].

Construction safety research is abundant and motivated by the alarming rates of accidents and fatalities, focusing on two perspectives: management and technology [2]. In general, workplace safety management is based on organizational and technological strategies. Construction safety standards and accident reduction are achieved through information and worker training, aiming to enhance the level of risk perception associated with the production process. However, the impact of traditional accident prevention strategies has been limited due to their reactive and regulatory nature [2,3]. A relevant aspect is the increased risks associated with the organization and production goals of construction companies.

According to Razi et al. [4], Artificial Intelligence (AI) is a broad field of computer science concerned with developing intelligent robots capable of performing tasks that traditionally require human intellect. In a more in-depth analysis, the same authors list the most common sub-areas of AI applicable in the construction sector, such as machine learning, computer vision, automated planning and scheduling, robotics, knowledge-based systems, natural language processing, and optimization, listing their advantages and disadvantages. AI plays a crucial role in assisting construction supervisors in minimizing accidents, supporting project efficiency, and significantly improving operational safety. Alongside the advancement of information and communication technology, various innovative technologies have been investigated to aid and improve existing management-driven safety management practices. Besides the aid of technologies, new injury prevention strategies have been developed for the construction industry. The risk analysis method is one of them, used in safety programs to improve safety performance. A relevant factor is the relationship between the type of construction project and the type of accident.

Data mining methods are applicable in various fields dealing with different types of data and objectives. Studies focusing on DM techniques applied to construction safety date back to no later than 2014 [5]. Our study has been developed as part of Inail’s 2022–2024 research program and the objective “Study of the effectiveness and efficiency indices related to innovative technologies aimed at preventing the risk of injury in highly variable work environments”. Considering the articles found to be eligible for review (Appendix A), we first focused on data mining methods (Appendix B) by categorizing them into three types: supervised, unsupervised, and other (not supervised). Subsequently, through cluster analysis, principal component analysis, and meta-analysis, we identified statistical associations between the two types of methods and the study objectives, types, and sources of data. The protocol of review is led by the Preferred Reporting Items for Systematic Reviews and Meta-Analyses (PRISMA) protocols [6]. Despite its limitations, the review has enabled us to determine the most effective method between supervised and other methods for different survey purposes, sources, and data types. It also gives a reference for those who have to choose and apply a DM method on the basis of certain fundamental inputs, such as the type of data available and the objectives to be achieved.

Section 1 of the article introduces the background and objectives of the investigation. Section 2 describes the materials and methods, while Section 3 presents the results obtained from applying cluster analysis, PCA, and meta-analysis. Section 4 offers an extensive discussion of the results considering the current state of the art and our future goals. Finally, Section 5 summarizes the salient results achieved in this review.

## 2. Materials and Methods

The set of articles published from 2014 to September 2023, which were useful for the purposes of this review, was extracted from Scopus [7,8] and ProQuest [9]. Authoritative sites on conferences in the field of computer science and DM and Management in Construction field were queried; however, only Web of Conference provided an eligible contribution for the purposes of our review.

### 2.1. Selection and Inclusion Criteria

All searches were conducted using a combination of subject headings and free-text terms. The search criteria applied in the PRISMA methodology were obtained by successive reiterations using different arguments and different Boolean AND and OR operators. Of these reiterations, the final one is given in Appendix A. We focused exclusively on peer-reviewed articles, conference papers, and book chapters. The topics included were “machine learning” AND construction AND work OR safety, across the following subject areas: (i) Engineering, (ii) Social and Environmental Sciences, and (iii) Computer Sciences. The criteria applied in the search strategy are defined in Table 1. The final search strategy was developed through several preliminary searches, including (i) articles, (ii) conference papers, and (iii) book chapters (Appendix A).

**Figure 1:**
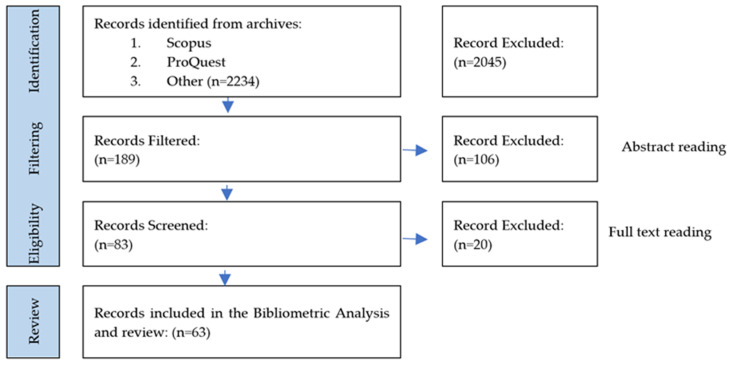
PRISMA criteria for the selection of documents and eligibility flowchart. Source: Scopus and ProQuest data. Years: 2014 to September 2023.

Figure 1 summarizes the result of the PRISMA document selection process. The collected dataset includes information on authors, title, year of publication, source of title, volume, issue, number of pages, citation number, DOI, affiliations, author information, abstract, keywords, type of publication, and further information. Three authors (AP, AB, and DB) independently reviewed the titles and abstracts to assess the eligibility of all studies. We applied PRISMA procedures and checklists [6] to identify topic datasets and keywords and filter content according to the abstract, assessing the eligibility of publications in the research scope. Further insights were made into the selected articles by conducting full-text reviews and analyzing the content for search purposes (see Figure 1) [10]. Disagreements were resolved by a fourth evaluator (ML) until a consensus was reached between the authors. Only studies that met the eligibility criteria were included.

### 2.2. Risk of Bias for Selected Studies

The risk in non-randomized studies was assessed based on the following biases: (1) due to confounding, (2) in the selection of the types of data in the study, (3) in the classification of the study objective, (4) due to missing data, (5) in the measurement of outcomes, (6) in the evaluation metrics, and (7) in the selection of the reported outcome. Each individual study included was assessed as having a low, moderate, severe, and critical risk of bias. If critical information was missing for the assessment of the risk of bias, these studies were considered devoid of information.

### 2.3. Data Quality and Items

The titles and abstracts of the identified studies were independently checked at two different points in time. Eligibility and inclusion criteria were initially assessed on a subset of 30 studies before searching all databases. Decisions were made by examining both the abstracts and the full texts. Only studies that were complete and met all inclusion criteria were included in the qualitative and quantitative synthesis. The information and data included in the papers obtained through the PRISMA method were then included in the review.

### 2.4. Study Design

The scientific articles falling under the eligibility criteria of PRISMA were pre-processed to extract information suitable for the purpose of review. The 63 papers included in the review were categorized by 31 source titles and publication year. The bibliometric analysis involved a review of the global literature and geographic mapping worldwide. The cluster analysis (HC) was used to find the best aggregations between groups. Using the Silhouette index, it was found that the best degree of aggregation was represented in a cluster plot based on correlations and variances. Principal component analysis (PCA) was useful to find the correlation classes between the various parameters of the dataset in a simplified reading of the results. Through PCA, we reduced the items and obtained the extent of correlation between variables, methods, and components. The meta-analysis of these classes was useful in estimating the reliability of HC and PCA results and the odds ratios OR and confidence intervals CI of groups of items. Spatial data collection, analysis, classification, and bibliometric analysis were performed with VOS viewer [11], R (https://www.r-project.org/ accessed on 13 June 2024), and QGIS 3.18.3-Zürich software, (Free Software Foundation, Inc., Boston, MA 02110-1301 USA).

**Figure 2:**
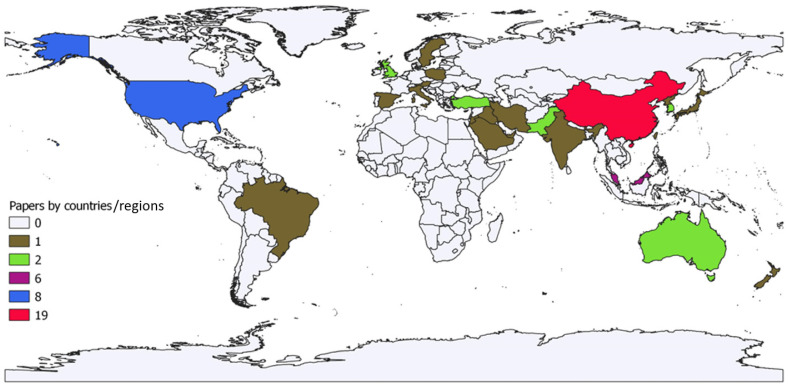
Map of papers included in the review by country/region. Years: 2014 to September 2023. Source: Authors’ processing from Scopus and ProQuest data. QGIS 3.18.3.

Articles that met the PRISMA eligibility criteria were classified according to their country/region of origin (corresponding author) and placed in one of the classes depicted in Figure 2 according to the numerosity marked by a colour (brown, green, purple, red, blue). 

In Figure 2, to the brown class belong the 18 countries/regions with 1 article such as Austria, Brazil, Cyprus, India, Iran, Iraq, Italy, Japan, Jordan, New Zealand, Poland, Republic of Korea, Saudi Arabia, Singapore, Spain, Sweden, Taiwan, United Arab Emirates. To the green class belong the 6 countries/regions with 2 items such as Australia, Hong Kong, Pakistan, South Korea, Turkey, UK. To the purple class belong Malaysia with 6 items. To the blue class belong the USA with 8 items. Finally, to the red class, the most numerous, belongs China with 19 items. The details of classes 1, 2, 6, 8, 19 in Figure 2 and their articles are specified in Appendix B.

## 3. Results

### 3.1. Study Selection and Bibliometric Analysis

The search strategy based on the PRISMA eligibility criteria yielded 63 papers that were included in the review and categorized by 31 source titles (Table 2) and publication year. Regarding the latter, there was an increasing trend in publication from 2014 to September 2023, where the articles recorded the following trend: 1 paper each in 2014 and 2015, 3 papers in 2016 and 2017, 4 papers in 2019, 6 papers in 2020, 3 papers in 2021, 17 papers in 2022, and 23 papers in 2023. 

### 3.2. Classes of Data and DM Methods

The information extracted from the individual articles was grouped into six homogeneous classes: DM method, study objective, field, data type, DM type, and resource type. In a separate dataset, we compiled the study objective, type of data under investigation, applied DM methods, applied DM type (supervised, unsupervised, and other), validation metrics (if available), the DM method found to be most effective, and number of rows and columns in the dataset used by the authors (if available). The set of classes has been reduced to 20 features, which are summed up in Appendix D and Appendix F. The 63 selected articles provided 206 observations, 89 DM methods (50 of which were considered the best method), 4 survey purposes, 3 fields, 7 data types, 3 DM types, and 3 resource types (as detailed in Table 3 and Appendix D and Appendix F). DM method: for each method and each method found to be the most effective (best method among those applied by the authors), absolute frequencies were reported. This feature consists of the method(s) used by the authors (from 1 method to more than 10). Study objective: this feature consists of the purpose for which the authors applied one or more methods in their article (from 1 up to 4). As a survey objective, we obtained X1 classifying (18%), X2 decision making (15%), X3 monitoring (16%), and X4 predicting (51%). Field: this characteristic indicates the source from which the data came in terms of construction process data, accident data, and health and safety risk management data. As fields, we obtained X5 construction process (38%), X6 occupational accident (34%), and X7 health and safety risk management process (28%). Data type: this means the format in which the information is represented and made available to authors for research purposes. The types of data investigated were X8 construction project (5%), X9 institutional dataset (70%), X10 interview report (2%), X11 literature data (3%), X12 narrative text (6%), X13 signal (10%), and X14 simulation (4%). DM type: this indicates a grouping into three classes of the feature DM method. The need for this additional class is linked to the fact that some DM methods can be used both as supervised and unsupervised. As the Type of DM investigated, we found X15 supervised method (58%), X16 unsupervised method (24%), and X17 other method (18%). Resource type: this feature was necessary to specify the field to which the authors’ results referred. This is the case with data from accidents to predict the outcome of a production process. As a resource type, we found X18 process (63%), X19 environment resource (15%), and X20 plant and machinery resource (22%).

### 3.3. Cluster Analysis

Clustering is a significant approach in DM that aims to identify groups within datasets. In real-world applications, both numeric and categorical features are often used to define the data. Clustering analysis is one of the most important approaches in DM, and it seeks to find the nature of groupings or clusters of data objects within an attribute space [72,73,74]. For an exploratory approach, we applied clustering analysis to the dataset in Appendix D. With this unsupervised ML approach, the algorithm processes input data and generates a sequence of clusters based on relational similarities with surrounding data points. The questions to answer in this DM method are “when do we stop combining clusters?” and “How do we represent clusters?”. By applying hierarchical clustering (HC) and the appropriate indexes, we identified the optimal number of clusters of our data.

**Figure 3:**
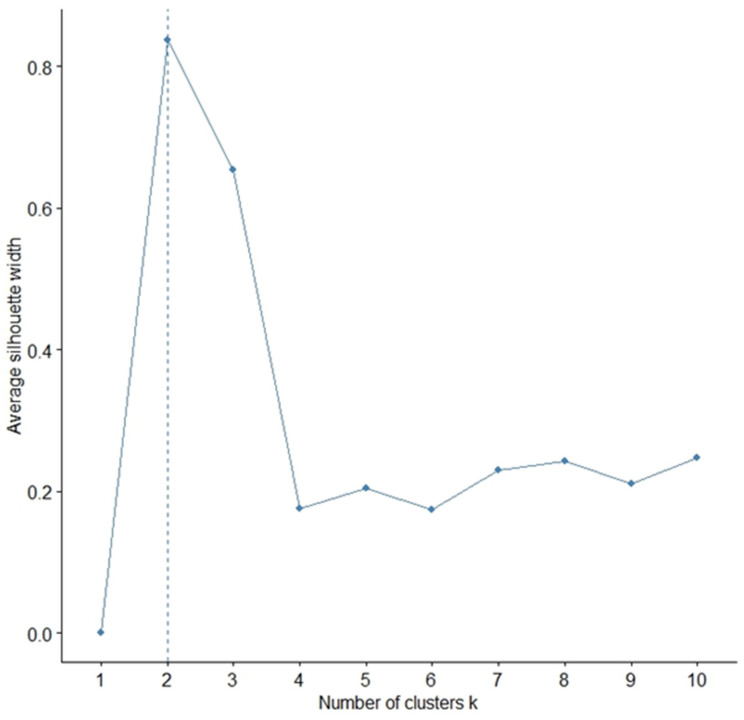
Silhouette test. Representation of optimal number of clusters. Years: 2014 to September 2023. Source: Authors’ processing from Scopus and ProQuest data.

According to Chang and Mirking, the “silhouette” index provided the best determination of cluster number; the highest average silhouette width indicates the optimal number of clusters. The concept of silhouette width involves the difference between the within-cluster tightness and separation from the rest. Specifically, the silhouette width *s_i_* for entity *i* ∈ *I* is defined as:

(1) $$s_{i}=\frac{b_{i}-a_{i}}{max(a_{i},b_{i})}$$

where “$a_{i}$” is the average distance between “*i*” and all other entities in the cluster to which “*i*” belongs, and “$b_{i}$” is the minimum of the average distances between “*i*” and all entities in every other cluster. Silhouette width values range from −1 and 1. If the silhouette width value for an entity is approximately zero, it means that the entity could also be assigned to another cluster. If the silhouette width value is close to −1, it means that the entity has been incorrectly classified. If all silhouette width values are close to 1, it means that the set “*i*” is well clustered [75]. As shown in Figure 3, the best aggregation of the dataset in Appendix D consists of two clusters with a silhouette index of more than 0.7.

**Figure 4:**
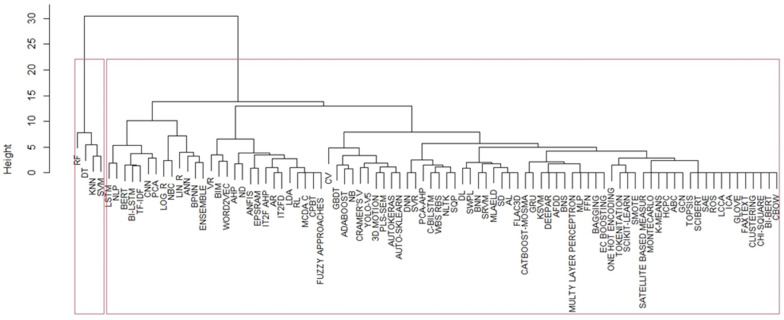
Hierarchical cluster dendrogram. Euclidean distance (height) vs DM methods and cluster (“Ward.D2”). Years: 2014 to September 2023. Source: Authors’ processing from Scopus and ProQuest data.

We created the item groupings through an iterative hierarchical process of aggregating pairs of “most similar” groups of methods by calculating the dissimilarity (“distance” for triangular inequality). Thus, we obtained the dendrogram in which the Euclidean distance between the elements, the similarity, and the shape of the clusters are represented. Figure 4 shows the results of the hierarchical cluster (HC) for the dm methods included in Appendix C and Appendix D. The abscissa shows the dm methods, the ordinate the Euclidean distances between the methods. The two red squares comprise the two large clusters into which the dm methods have been aggregated according to their Euclidean distances. Specifically, the first box explains the first cluster, containing RF (random forest), DT (decision tree), KNN (k-nearest neighbour) and SVM (support vector machine). The second large box contains the grouping of the remaining methodologies.

### 3.4. Principal Component Analysis (PCA)

The objective of PCA is to identify suitable Y linear transformations of the observed variables that are easily interpretable and capable of highlighting and synthesizing the information inherent in the initial matrix X. This tool is particularly useful when dealing with a considerable number of variables from which one wants to extract as much information as possible while working with a smaller set of variables [73,74]. The analysis was carried out on the data matrix that contains 89 individuals corresponding to DM methods and 22 quantitative variables (Appendix D).

(2) $$X={\left(X_{1},X_{2}\ldots X_{p}\right)}^{T}$$

Given a matrix *X* containing *n* features, it is possible to obtain a matrix of new data *Y*, consisting of *p* interrelated variables, which turn out to be linear combinations of the first. Each principal component can be expressed as follows:

(3) $$\begin{array}{c} \to \\ Y \end{array}=\left[\begin{array}{c} Y_{1}\\ \vdots \\ Y_{p} \end{array}\right]=\left[\begin{array}{ccc} l_{11} & \cdots & l_{1p}\\ \vdots & \ddots & \vdots \\ l_{p1} & \cdots & l_{pp} \end{array}\right].\left[\begin{array}{ccc} X_{11} & \cdots & X_{1p}\\ \vdots & \ddots & \vdots \\ X_{p1} & \cdots & X_{pp} \end{array}\right]$$

(4) $$\begin{array}{c} \to \\ Y \end{array}=l_{ij}X_{1}+l_{ij}X_{2}+\ldots l_{ip}X_{p}\;where\;i=1,2\ldots p$$

The generic coefficient $l_{ij}$ is the weight that the variable *Xj* has in finding the principal component *Y_i_* (with *i =* 1, 2, *k*, *p*) [11,38]. The larger $l_{ij}$ is (in absolute value), the greater the weight that the values *X_j_* (*j =* 1, 2, *k*, *p*) have in deciding a given principal component [74]. The data extracted from the articles included in the review were organized into subclasses (Table 3) and grouped according to Appendix D. The linear correlation coefficients *l_ij_* between each pair of standardized variables included in Appendix E are the result of the ratio of the covariance to the product of the standard deviation between *x_i_* and *x_j_* (*l_ij_ = σ_ij_/σ_i_σ_j_*). The Pearson correlation coefficient *l_ij_* provides the intensity and direction of the linear relationship between the variables. The bold numbers express the significance of the correlation given by *p*-values below 0.05 (Appendix E). Before conducting PCA, we checked the linear relationship, the correlation between all quantitative variables, and the absence of outliers [74]. The correlation matrix suggested the features in Appendix D be grouped for a more effective PCA. Proceeding with successive reiterations of the correspondence of different aggregations of features, we obtained the corresponding performances of the PCA. The tables and images in this paragraph refer to the performance found more concise and consistent with the results of the cluster analysis.

#### 3.4.1. Inertia Distribution

**Figure 5:**
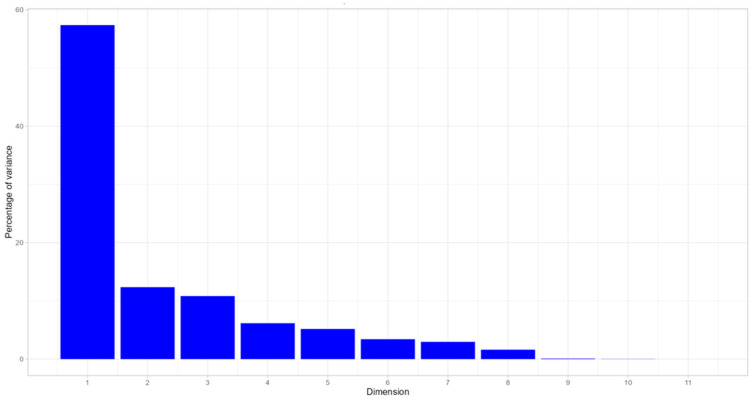
Decomposition of the total inertia by axes. Dimension vs. percentage of variance.

The dataset contains 89 individuals corresponding to DM methods and 20 features. Analysis of the graphs reveals no outlier. The inertia of the first dimension shows whether there are strong relationships between variables and suggests the number of dimensions that should be studied. The first two dimensions of analysis express 69.71% of the total dataset inertia; that means that 69.71% of the individual (or variable) cloud total variability is explained by the plane. This percentage indicates that the first plane effectively represents the data’s variability. The first factor is the main one: it expresses 57.36% of the variability of the data (Figure 5).

In this case, the variability relating to the other components may be less significant despite the high percentage. The first axis has a higher amount of inertia than the 0.95 quadrant of the random distribution. This observation suggests that only two axes carry information. Consequently, the description will stick to these axes.

The criteria for selecting dimensions in the final model are threefold: the Kaiser rule where eigenvalues are greater than 1 (Table 4); the proportion of variance explained by the components at least equal to 60–80% of the overall variability (Table 4); and the Cattell rule, according to which the right number of components corresponds to the elbow or change in slope in the component–eigenvalue graph (Figure 5). From these observations, it could be better to also interpret the dimensions as greater or equal to the second one. The above criteria allowed us to assign a “label” to each component.

#### 3.4.2. Axes Descriptions

Dimension 1 opposes individuals such as dt (32), knn (49), svm (81), and rf (69) to the right of the graph characterized by a strongly positive coordinate on the axis to individuals such as MCDA C (58), characterized by a strongly negative coordinate on the axis (to the left of the graph).

**Figure 6:**
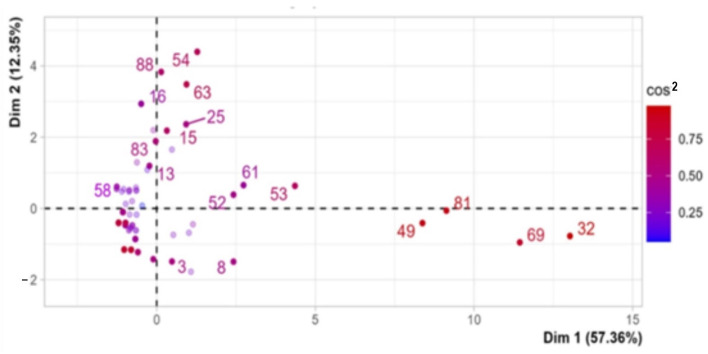
PCA. The graph of individual (DM methods). Dim1 vs. Dim2 (correlation or cos^2^ > 0.4).

Dimension 2 opposes individuals such as lstm (54), word2vec (88), nlp (63), and BIM (16), which are located at the top of the graph and characterized by a low positive coordinate on the axis, with individuals such as ann (8), adaboost (3), which have low negative coordinates on the axis and are located at the bottom of the graph (Figure 6).

Dim1 group 1 (dt, knn, svm, and rf) shares high values for the variables “predicting”, “supervised”, “monitoring”, “frequency”, “institutional data”, “data project-simulation-signal”, “classifying”, “best method”, and “interview-literature-text” (variables are sorted from the strongest to the weakest). Group 2 is characterized by a negative coordinate on the axis, with the individual MCDA C (58) sharing low values for the variables “interview-literature-text”, “classifying”, “frequency”, “institutional data”, “monitoring”, “predicting”, “supervised”, “project-simulation-signal”, “best method”, and “other methods” (variables are sorted from the weakest to the strongest). The variables “supervised” and “frequency” are highly correlated with this dimension (correlations of 0.94 and 0.98, respectively). These variables could therefore be summarized as dimension 1. Dim2 group 1 shares high values for the variables “not supervised” and “decision making” while group 2 shows the same for “monitoring”, “machinery”, and “environment” (Table 5 and Table 6).

According to the correlation method variable and axes, the x-axis (Dim1) can be renamed “Supervised methods” (dt, knn, svm, and rf) applied to institutional data to classify and make inferences (predicting)”. The y-axis (Dim2) can instead be renamed “Not-supervised methods” (lstm, word2vec, nlp, and BIM) applied to project, simulation signal, interviews, literature, or textual data to make decisions and classify.

### 3.5. Meta-Analysis

**Figure 7:**
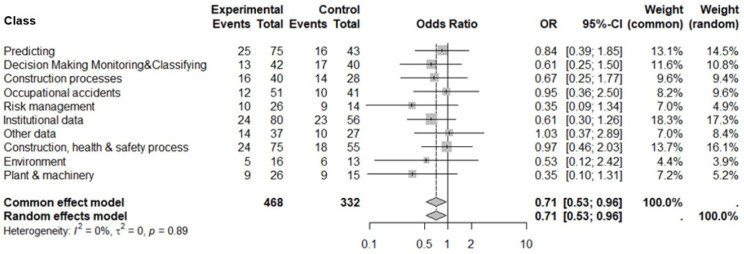
Funnel plot. Odds ratio (OR) and relative confidence interval (95% CI) for the total number of data mining methods analyzed, by class. The relative weight of each estimate in the analysis is marked with a box. The diamond represents the meta-analytical OR. Years: 2014 to September 2023. Source: Authors’ processing from Scopus and ProQuest data. R.

The data from the complete collection of studies selected according to the PRISMA method and aggregated according to the classes defined in Table 1 allowed us to derive a single conclusive result that answered our research question. Through a meta-analysis, we assessed whether supervised methods were more effective than not-supervised ones across the various classes. The forest plot summarizes the results of the meta-analysis, which include the OR with its CIs, the sample size weight, the heterogeneity of the data, and a quantitative, whole-data assessment of the effectiveness of the treatment with supervised methods (Figure 7).

The heterogeneity is null (the sets under study are compatible). The analysis of the groups shows that the CIs intercept the “no effect” line and lose significance when taken individually; however, they consistently overlap and are similar to each other. Figure 7 shows a generally positive trend toward data treatment with supervised methods (on the left from the “no-effect” line), summarized by OR = 0.71 and the CI (0.53–0.96).

## 4. Discussion and Future Directions

Studies focusing on DM techniques applied to the construction industry are recent, dating back to 2014 at the latest and, therefore, the review dates we reviewed were from 2014 to September 2023. The number of articles in this sector increased from 1 in 2014 to 23 in 2023. Similarly, the evolution of the total number of applied DM techniques increased from 5 between 2014 and 2016 to approximately 60 in 2023 (data not yet completed at the time of the survey).

In the construction process field, 20 out of 63 observations were made regarding the construction of buildings, dams, roads, and tunnels. Within this field, 60 out of the 206 observations covered topics such as construction delays [38]; crane, drilling, and excavation tasks [13,21,24,41,44,45,50]; geological conditions [55]; scaffolding collapse [51]; transport delays [56]; tunneling [19,26,27,37,42,58,70]; and worker and machinery location [43,71]. According to Erzaij et al., project suspensions are among the most persistent challenges facing the construction sector due to the difficulty of the industry and the essential interdependence between the bases of delay risk. The influence of delays can lead to increased time, costs, disputes, litigation, and overall rejection. The study aims to develop a data prediction tool to examine and learn the sources of delay based on previous data from construction projects, using decision trees and Bayesian naïve classification algorithms. Among the prediction models developed by the authors applied to 97 projects, the decision tree showed the highest accuracy [38]. Kumari et al. [71] investigated a machine learning architecture for excavator position detection using a Global Positioning System (GPS), which can guarantee an excavator and driver position remarkably close to the real one. Wang J. et al. [27] used the principal component analysis (PCA) approach to select input factors for the prediction of tunnel-boring machine (TBM) performance, particularly the travel speed. Liu et al. [15] developed a model capable of predicting tunnel-boring machine disc replacements based on a binary classification algorithm of the Gaussian kernel support vector type cutting performance. After being trained using a period of historical data, the proposed model can predict whether cutter disc replacement is necessary, thus reducing the time required for periodic inspections. Lin et al. [43] investigated the feasibility of a real-time location service system using the Wi-Fi fingerprinting algorithm for the safety risk assessment of tunnel workers. A location algorithm based on signal strength (RSS) and an artificial neural network (ann) were used for location analysis and risk assessment. Wei [51] developed wind speed prediction models based on various deep learning and machine learning techniques, in particular deep neural networks, neural networks with short-term memory, support vector regressions, random forests, and k-nearest neighbors. Subsequently, the author analyzed the wind force on the scaffold and assessed the probability of the scaffold collapsing under the action of the wind.

In the Occupational accident field, 16 out of 63 papers dealt with data on accidents and injuries at work from 2014 to 2023. In the class “occupational accidents,” 89 out of 206 observations covered the following topics: reporting of accidents [3,12,14,16,17,22,23,25,26,28,29,40,46,52,59] and days away from work. On this topic, Yelda et al. analyzed textual narratives to predict injury outcomes and days off work in a mining operation. For this purpose, they used decision trees, random forests, and ANNs, and the performance of these models was compared with that of logistic regression [47]. Lee et al. [16] proposed an optimized data preprocessing method to minimize the variables and main elements in diverse and complex work accident data and built an ML prediction model to achieve this. Specifically, they analyzed the correlations using a flood flow diagram and applied clustering and principal component analysis (PCA) to analyze the relationships between the main variables and to draw broader conclusions. However, accidents are unevenly recorded in narrative form. Construction accident reports hold a wealth of empirical knowledge that could be used to better understand, predict, and prevent the occurrence of accidents in the construction sector. Large construction companies and federal agencies, such as the Occupational Safety and Health Administration (OSHA), hold these reports in the form of huge digital databases [17]. Zhang J. et al. [17] utilized accident narrative data obtained from the official OSHA website, presenting a new unified architecture with a bi-directional short-term memory model (BiLSTM) and a convolutional layer for the classification of construction accident causes. Tixier et al. [22] and Zhan F. et al. [25] proved how the study of safety attributes and outcomes can be automatically and accurately processed from unstructured accident reports using natural language processing (NLP).

In the risk management field, 25 out of 63 papers dealt with data on the “risk management process”. In this class, 54 observations out of 206 concerned the following topics: awkward working postures [20,30]; compliance with Health and Safety standards [31,32,64]; risk assessment [2,4,48,57,60,65,68]; safe climate [33]; slope instability [18,63]; teaching–training tasks [34,49,62]; unsafe behaviors [35,36]; worker fatigue—heat stress [24,39,69]; and site image [66,67]. Antwi-Afari et al. [20] used deep learning networks to automatically extract relevant features with spatial-temporal dependence acquired by a wearable insole pressure system. The aim was to use deep learning-based networks and sensor data from wearable insoles to automatically recognize and classify types of awkward working postures for construction workers. So, they adopted recurrent neural networks (RNNs) and deep learning models to train time series of plantar pressure data acquired from a wearable insole pressure sensor. Wang F. et al. [60] provided a strategic view of the relationships between different organizational objectives and technical risks that may arise during the construction of a tunnel. They created a systems-based model integrating Systems Dynamics (SD), Bayesian Belief Networks (BBNs), and Smooth Relevance Vector Machines (sRVMs) called the Organizational Risk Dynamics Observer (ORDO). The model was applied to an urban metro project built in Wuhan, China, and was used to provide guidance on effective accident prevention strategies. Mostofi et al. [3] explored the predictive ability of a multilayer GCN algorithm that learns the connection between construction accidents and project types, believing that richer information from existing safety and construction accident datasets by project type would provide better learning for the predictive model adopted. In addition, it would have supplied more information to predict the severity of accident consequences. The authors proved the effectiveness of the network representation of construction accidents in improving the learning capability of the ML model by using a feedforward reference network (FFN) algorithm with parameters like those used in the GCN algorithm to predict severity outcomes. The use of prefabrication is attracting increasing interest in the construction industry due to sustainability aspects, product quality, high production efficiency, and cost-effectiveness. Dealing with this topic, Zhu and Liu [68] developed a prediction and risk assessment model related to the supply chain management of precast buildings. The BP neural network can be used to predict the risk of the prefabrication supply chain.

Soil instability and landslides are major problems in the construction sector that can lead to safety risks for workers and the public, but also to considerable economic damage due to work stoppages. In this regard, Bay et al. [18] evaluated 102 cases of slopes with arc-shaped failure modes using eight machine learning regression methods. The slope safety factor prediction models were set up by performing cross-validation and hyper-parameter adjustment of the model. Furthermore, based on objective weighting and TOPSIS methods, a model was developed to evaluate the performance of the machine learning model and find the best FOS prediction model. Sadeghi et al. [48] developed an Ensemble Predictive Safety Risk Assessment Model (EPSRAM) to assess the health and safety risks of workers on construction sites based on the integration of neural networks and fuzzy inference systems. The model introduces innovation in countries/regions such as Malaysia, where there is continued growth in the construction industry but where there is a lack of studies on OHS assessments of workers involved in construction activities. Such circumstances may expose construction workers to the risk of developing fatigue. If workers continue to work under fatigued conditions, they are prone to the development of work-related musculoskeletal disorders (MSDs). Yu et al. [24] and Yan et al. [69] developed a combination of computer vision technology and biomechanical analysis for non-intrusive whole-body fatigue monitoring of construction workers using 3D model data from the motion capture algorithm and biomechanical analysis.

Zhao et al. [62] conducted a study on efficient and parallel DM and machine learning methods and algorithms distributed on a large scale and proposed an experiential teaching model focused on the cultivation of independent learning ability and the subjective initiative of individual learners. The article, which could have been excluded for review, was nevertheless kept as it combined the importance and technical challenges of the algorithms themselves and the context of the practical application needs of the field. It reported research on methods and algorithms for DM and machine learning, distributed on a large scale for training purposes. As an innovative teaching model, the experiential teaching model described in it focuses, among other things, on cultivating individual learners’ independent learning ability and subjective initiative, which was found to effectively activate the atmosphere of the working class/environment and improve the teaching effect. It has been included as one of the articles that innovatively deals with the risk management process, including health and safety training in the workplace. Other studies, not included in the review, report analyses based on the effectiveness of combinations of Smart Construction Safety Technologies (SCSTs), potentially able to generate information useful for DM, and the measurement of the effectiveness of the same technologies, both alone and combined [76]. Zerman et al. used machine learning to create a predictive model to help detect the most likely factors that affect fatal accidents due to falls from heights in the construction industry in Malaysia. To this end, the authors used institutional data from the Malaysian Department of Occupational Safety and Health Records of Occupational Accidents and applied different machine learning models such as random forest (rf), gradient boosting (gbdt), logistic regression (lr), naïve Bayes (nbb), multilayer perceptron (mlp), and knn. The model obtained from the random forest application was the best [61].

Regarding the type of data used in DM, 39 out of 63 papers dealt with institutional datasets (2016–2023), 8 used signal and video data (2014–2023), 4 used narrative texts (2016–2022), 5 used construction projects (2016–2023), 4 used literature data (2020–2023), 3 used simulations (2015–2023), and 2 out of 63 used an interview report (2023). The data used may have different characteristics in reference to specific aspects of an occupational injury, such as, for example, the body parts affected and the expected probability. Other studies focus on the observation of environmental and meteorological precursors of accidents, e.g., associated with the collapse of scaffolding [19] and slope instability [18,63]. Liu et al. analyzed data from sophisticated and technologically innovative machine monitoring, capable of returning and processing geological data and faults and predicting the progress of TBM and maintenance, avoiding downtime and inspections [26]. According to Schindler et al. [70] and Leng et al. [42], the use of satellite data has proved to be a winning strategy compared to ground surveys. Data collected by sensors were used to assess the state of effort associated with the awkward working postures of workers while performing work on the construction site [20] or physical fatigue and workers’ heat stress [69]. Another interesting use of data involves the construction practitioner’s interview through which processes and occupational risk information are integrated [4].

According to Gondia et al., the factor that most determines delays in the construction sector is the late payment of the contractor. The authors used naïve Bayes and decision tree algorithms to predict project downtime. The decision tree showed an accuracy of approximately 89%, which is better than that obtained with naïve Bayes due to the type of data string. The model proposed by the authors has been applied to approximately 97 projects and has been found to have the potential to reduce delays and, consequently, also costs [53]. Shirazi and Toosi used the literature, interviews, and project data. According to Shirazi et al., delays in construction are among the most important challenges in the sector, especially in the infrastructure sector, where serious socio-economic consequences may occur. The authors identified 65 risk factors associated with delays through data derived from a comprehensive review of the literature and interviews and applied principal component analysis. The resulting dataset was used to develop a deep perceptron neural network (mlp-nn) model to predict project delays. The use of a deep-nn (dnn) model showed that the addition of characteristic project data to the training dataset significantly improved the prediction performance of deep-mlp. [54].

By focusing on health and safety aspects, quality, in terms of the homogeneity and standardization of the various sources of institutional accident data included in the review, can be affected by the different methods of acquisition, from one institution to another and from one country/region to another. It can also be assumed that the data produced by technologies and machines used in the processes have a higher degree of homogeneity and standardization than the former. Liu et al. underlined the significance of employing innovative and efficient safety management technologies, along with new management approaches and automated methods based on artificial intelligence, to promptly detect and eliminate risks. According to the authors, these innovative technologies would mitigate any deficiencies in site management, significantly improve site safety management, and eliminate risks at the source [26]. An increasingly widespread orientation towards automated management of the site or parts of it would not only lead to an improvement in the health and safety of the processes but also a significant improvement in the quality of the data coming from the construction field. It can be assumed that soon, accident data collection techniques will not be able to function without innovative technologies capable of automatically acquiring information on near misses, accidents, and injuries in the construction sector.

Intelligent technologies can generate a range of data that pertain to both the individual (e.g., worker) and the interaction and connection between different technologies. The Internet of Things (IoT) is gradually spreading in the construction sector, thus making an important contribution to the production of new data. Robots and collaborative robots play a significant role in technological innovation and data extraction as they can produce quality in terms of productivity, product quality, and the standardization of production processes. Furthermore, these technologies have the potential to produce high-quality data, which could play a significant role in the pre-processing of data required for the use of DM techniques. The use of these technologies in construction sites is still limited due to unresolved difficulties, attributable to the high variability of environmental conditions and the need to protect the secrecy of processes and the privacy of workers. Moreover, to accompany change, workers and enterprises need vocational training and management training [1].

Regarding the “construction processes”, “accidents”, and “risk management” fields, the results of the PCA are consistent with the literature analysis included in the review. The main component, D1, associates supervised methods such as dt, knn, svm, and rf with the prediction and classification of data without giving indications on the type of data and resource. The D2 main component instead associates not-supervised methods (unsupervised and others) with monitoring and decision making. D2 specifies not only the methods and objectives to be achieved but also the type and source of the data. Therefore, unsupervised methods like lstm, word2vec, nlp, and bim are associated with projects, simulations, signals, interviews, scientific literature, and texts. In addition, D2 combines these methods with data on work equipment (e.g., machines and installations) and the working environment (wind, temperature, geological stability, etc.). The two main components, D1 and D2 (Table 5 and Table 6), have the potential to guide the actors involved in the management of the data relative to yards. The latter concerns both construction processes and accidents and the management of health and safety risks on construction sites. Such evidence has been integrated with the results of the meta-analysis where better adaptability of the supervised methods is valued over those not supervised. The proposed approach has been useful in the association between methods (supervised and not) and data types, classified by type, source, field, and resources of the process from which the same data is derived. The study shows, among other things, part of the technological development present in construction yards that has been intercepted by the scientific literature. However, the study has limitations due to the very origin of the information analyzed. These are differences determined by the object of investigation that characterizes the individual papers included in the review. Another limitation is linked to the small size of the paper sample available for the survey, which could have a bearing on the significance of the results obtained. Any loss of significance of the data and the results obtained could also be attributed to the absence of standardized production protocols and the various levels of technology available on construction sites around the world, in the time frame of reference. In addition to these aspects, we also note the possible risks due to confusion and the correct classification of the type of data and the source, the objectives (predictive, monitoring, decision making, and classification) of missing data, and the extent of the results.

Reducing risks at the source is the most effective measure fpr managing health and safety at work. Unfortunately, it is not always possible to reduce risks at the source and, therefore, safety standards and the reduction in occupational accidents are achieved through prevention and protection measures (ILOSHA). An important aspect that is typical of construction companies is the increase in risks associated with organizational and production objectives. In this context, the use of advanced statistical and technological tools based on data mining can help, both in prevention measures and in measures to protect against occupational risks. The use of predictive techniques such as dt, knn, svm, and rf can be decisive in risk assessment, the key measure for risk prevention. Similarly, techniques such as lstm, word2vec, nlp, and BIM can aid in monitoring and decision making on site, for example, in the integrated management of safety and construction processes. Decision making can be applied to the choice of individual and collective prevention devices, the key measure of protection against occupational risks.

Further future development of the study should focus on a larger and more homogeneous sample of sources where the results are based on standardized and repeatable parameters resulting from data mining techniques. We believe that, despite the limitations of our work, the results obtained have added value to the complex problem of data mining in this sector.

## 5. Conclusions

Cluster analysis and PCA were applied to data from articles that met the PRISMA eligibility criteria and were included in the review. The study indicates an association between the types of methods used and objectives, scope, type of data, and resources under investigation. This association, based on correlation, was synthesized onto a single xy-plane (Dim1 and Dim2). The results of the PCA were consistent with those of the cluster analysis. Each of the two axes was assigned a label summarizing the significance of the entire review. The x-axis (Dim1) was labeled “Supervised methods (dt, knn, svm, and rf) applied to institutional data for classification and inference”. The y-axis (Dim2) was labeled “Not-supervised methods (lstm, word2vec, nlp, and BIM) applied to projects, simulations, signals, interviews, the literature, or textual data to classify and make decisions”. The meta-analysis, with an odds ratio (OR) of 0.71 and a confidence interval (CI) of 0.53 to 0.96, provides an overall estimate of the superior effectiveness of supervised methods compared to not-supervised ones.

## Figures and Tables

**Table 1:** Query input for document search inclusion criteria. Source: Scopus and ProQuest data.

Stream	Query
tit-abs-key	“machine learning” AND construction AND work OR safety
subject area	Engineering AND Social Science AND Environmental Science AND Computational Science
publication year	From 2014 to September 2023
document	Article, Conference Paper, Book chapter (Peer reviewed)
language	Not restriction

**Table 2:** Papers included in the review by source. Years: 2014 to September 2023. Source: Scopus, ProQuest data.

Source Title	Author	Papers
Accident Analysis and Prevention	[12]	1
Advances in Civil Engineering	[13]	1
Applied Sciences	[14]	1
Applied Sciences (Switzerland)	[15,16,17,18]	4
Applied Soft Computing	[19]	1
Automation in Construction	[20,21,22,23,24,25]	6
Buildings	[26,27,28,29,30,31,32,33,34,35,36]	11
Chinese Journal of Mechanical Engineering (English Ed.)	[37]	1
Civil and Environmental Engineering	[38]	1
Computer-Aided Civil and Infrastructure Engineering	[39]	1
E3S Web of Conferences	[40]	1
Engineering, Construction and Architectural Management	[41]	1
IEEE Access	[42,43]	2
IEEE Robotics and Automation Letters	[44]	1
International Journal of Computational Methods and Experimental Measurements	[45]	1
International Journal of Environmental Research and Public Health	[46,47,48,49]	4
IOP Conference Series. Earth and Environmental Science	[4,50]	2
Journal of Civil Engineering and Management	[51]	1
Journal of Safety Research	[52]	1
Journal of Construction Engineering and Management	[53,54]	2
Lecture Notes in Civil Engineering	[55,56]	2
Mathematical Problems in Engineering Volume	[2]	1
PLoS One	[57]	1
Rock Mechanics and Rock Engineering	[58]	1
Safety Science	[59,60,61]	3
Scientific Programming	[62]	1
Sensors (Switzerland)	[63]	1
Sustainability	[64,65,66,67]	4
Sustainability (Switzerland)	[3,68,69]	3
Visualization in Engineering	[70]	1
Wireless Communications and Mobile Computing	[71]	1
Total		63

**Table 3:** Classification of content included in the 63 articles selected by the PRISMA method. Years: 2014 to September 2023. Authors’ processing from Scopus and ProQuest data.

Class	n	Description	Index
dm method	89	Appendix C and Appendix D	
study objective	4	classifying, decision making, monitoring, predicting	x1–x4
field	3	construction process, occupational accident, risk management	x5–x7
data type	7	project, institutional data, interview, literature, text, signal & video, simulation	x8–x14
dm type	3	supervised, unsupervised, other	x15–x17
resource type	3	construction and h&s process, environment, plant and machinery	x18–x20

**Table 4:** PCA. Eigenvalues, percentage of variance, and cumulative percentage of variance.

Dim	Eigenvalue	% of Variance	Cumulative % of Variance
Dim1	6.31	57.36	57.36
Dim2	1.36	12.35	69.71
Dim3	1.19	10.83	80.54
Dim4	0.68	6.16	86.71
Dim5	0.57	5.18	91.88
Dim6	0.38	3.42	95.30
Dim7	0.33	2.97	98.27
Dim8	0.18	1.62	99.89
Dim9	0.01	0.09	99.97
Dim10	0.00	0.03	100.00
Dim11	0.00	0.00	100.00

**Table 5:** PCA. Axes descriptions and correlations between axes, methods, and variables (cos^2^ > 0.4). Years: 2014 to September 2023. Source: Authors’ processing from Scopus and ProQuest data. R.

Axes	(+)	(−)	DM Class	Study Objective	Data Type	Resource Type
Dim1	dt (32), knn (49), svm (81) and rf (69)	MCDA C (58)	supervised	classifying predicting	institutional data, interview-literature-text	-
Dim2	lstm (54), word2vec (88), nlp (63), BIM (16)	ann (8), adaboost (3)	other- supervised (not-supervised)	decision making monitoring	project-simulation-signal; interview-literature-text	machinery environment

**Table 6:** PCA. Axes descriptions, correlations between methods, and axes. Years: 2014 to September 2023. Source: Authors’ processing from Scopus and ProQuest data. R.

Dim1	Correlation (cos^2^)	*p*-Value	Dim2	Correlation (cos^2^)	*p*-Value
frequency	9.874 × 10^−1^	1.603 × 10^−71^	other type	8.413 × 10^−1^	5.790 × 10^−25^
supervised	9.694 × 10^−1^	7.675 × 10^−55^	decision making	5.077 × 10^−1^	3.801 × 10^−7^
institutional data	9.412 × 10^−1^	8.809 × 10^−43^	interview-literature-text	4.688 × 10^−1^	3.593 × 10^−6^
predicting	9.361 × 10^−1^	2.984 × 10^−41^	classifying	3.060 × 10^−1^	3.547 × 10^−3^
classifying	8.181 × 10^−1^	1.286 × 10^−22^			

## Appendix A

**Table A1:** Paper identification criteria, subject, and number (extraction: 20 March 2023 and 12 September 2023).

Subject	n.
“machine learning”	526.288
“machine learning” AND work	122.958
“machine learning” AND work AND safety	11.752
“machine learning” AND construction AND work OR safety	2.280
subject area	2.234
abstract reading	189
full text reading	81
selected papers	63

## Appendix B

**Table A2:** Papers by country/region. Classes as represented in Figure 2 (1: brown, 2: green, 6: purple, 8: blue, 19: red). The country/region references are shown in square brackets. Years: 2014 to September 2023. Source: Authors’ processing from Scopus and ProQuest data. QGIS 3.18.3

Country/Region	Class
Austria [55], Brazil [57], Cyprus [65], India [71], Iran [54], Iraq [38], Italy [58], Japan [44], Jordan [31], New Zealand [69], Poland [14], Republic of Korea [13], Saudi Arabia [66], Singapore [12], Spain [45], Sweden [25], Taiwan [51], United Arab Emirates [64].	1
Australia [34,59], Hong Kong [24,37], Pakistan [32,33], South Korea [16,21], Turkey [3,29], United Kingdom [20,70].	2
Malaysia [4,46,48,49,61,67].	6
United States [22,23,30,40,47,52,53,56].	8
China [2,15,17,18,19,26,27,28,35,36,37,41,42,43,50,60,62,63,68].	19

## Appendix C

**Table A3:** DM methods included in the review. Source: Authors’ processing by Scopus and ProQuest archives, R.

DM Method	DM Method Description	DM Method	DM Method Description
abc	approximate bayesian computation	it2f–ahp	interval type-2 (IT2) fuzzy-analytic hierarchy process
adaboost	adaptive boosting (ensemble)	it2fd	interval type-2 (IT2) fuzzy Delphi
afdd	automated fault detection and diagnostics	k-means	k-means clustering
ahp	analytic hierarchy process	knn	k-nearest neighbour
al	ml-based active learning framework	ksvm	support vector machines in kernlab
anfis	adaptive neuro-fuzzy inference system	lcca	ml based life-cycle cost analysis
ann	artificial neural network	lin r	linear regression
ar	augmented reality	log r	logistic regression
autokeras	automl system based on keras	lstm	long short-term memory
auto-sklearn	automatic scikit-learn	mlaeld	machine learning architecture for excavators’ location detection
bagging	bootstrap aggregating	mlp	multilayer perceptron
bert	bidirectional encoder represent. for transformers	monte carlo	montecarlo method
bi-bert	binarized bidirectional encoder represent. for transformers	mcda-c	multicriteria methodology for decision aiding-constructivist
bi-lstm	bi-directional long short-term memory	mosma	multi-objective slime mould algorithm
bim	building information modeling	nb	naïve bayes
bnn	binarized neural network	nbc	naive bayes classifier
bns	bayesian networks	nlp	natural language processing
bpnn	back propagation in neural network	nltk	natural language toolkit
catboost	gradient boosting on decision trees	onehotencoding	onehotencoding in scikit-learn
c-bilstm	convolutional bi-directional long short-term memory	pca	principal components analysis
cbow	continuous bag of words	pca-ahp	analytic hierarchy process-principal component analysis)
chi-square	chi-square	pls-sem	partial-least-squares structural-equation modeling
clustering	clustering	rf	random forest
cnn	convolutional neural network	rl	reinforcement learning
cpbt	cognitive psychology and bloom’s taxonomy	ros	robot operating system
cramer’s v	cramer’s v	sae	sparse autoencorder
cv	computer vision process	satellite-based meas.	satellite-based measurements
deepar	autoregressive recurrent networks	scibert	scientific bidirectional encoder represent. for transformers
dl	deep learning	scikit-learn	key library for pyton programming language
dnn	deep neural network	sd	system dynamics
dt	decision tree learning	swpl	smart work package learning
ebt	ensemble of boosted tree	smote	synthetic minority over-sampling technique
ensemble	ensemble	sqp	sequential quadratic programming
epsram	ensemble predictive safety risk assessment model	srvm	smooth relevance vector machines
faxtext	faxtext	svm	support vector machines
ffn	feed-forward neural network	svr	support vector regression
flac3d	flac3d	tf-idf	term frequency-inverse document frequency
fuz	fuzzy approaches	tokenitation	split sentences into small units
gbdt	gradient boosted decision trees	topsis	technique for order of preference by similarity to ideal solution
gcn	graph convolutional networks	3d motion	3d motion
glove	global vectors for words representation	vr	virtual reality
gru	gated recurrent unit (recurrent neural network)	wbs-rbs	work breakdown structure-resource breakdown structure
hcpc	hierarchical clustering on principal components	word2vec	word2vec (nlp)
ica	independent component analysis	yolo-v5	you only look once

## Appendix D

**Table A4:** DM method by frequency (F), best method (BM), and variable (Matrix X). x1–x4 Study objective (classifying, decision making, monitoring, and predicting); x5–x7 Field (construction processes, occupational accidents, and risk management); x8–x14 Data type (project, institutional data, interview, literature, text, signal and video, and simulation); x15–x17 DM type (supervised, unsupervised, and other); x18–x20; Resource type (construction and H&S processes, environment, plant, and machinery). Source: Authors’ processing by Scopus and ProQuest archives and R.

Method	F	BM	x1	x2	x3	x4	x5	x6	x7	x8	x9	x10	x11	x12	x13	x14	x15	x16	x17	x18	x19	x20
abc	1	1	0	0	0	1	1	0	0	0	1	0	0	0	0	0	0	1	0	0	0	1
adaboost	3	0	0	0	1	2	1	1	1	0	3	0	0	0	0	0	3	0	0	1	1	3
afdd	1	1	0	0	0	1	0	0	1	0	1	0	0	0	0	0	1	0	0	1	0	1
ahp	2	2	0	1	0	1	0	1	1	0	2	0	0	0	0	0	0	2	0	0	1	2
al	1	1	0	0	0	1	1	0	0	1	0	0	0	0	0	0	0	0	1	1	0	1
anfis	1	1	0	1	0	0	0	0	1	0	0	0	1	0	0	0	1	0	0	1	0	1
ann	5	4	0	0	0	5	4	1	0	0	4	0	0	0	1	0	5	0	0	1	1	5
ar	1	0	0	1	0	0	0	1	0	0	0	0	0	0	0	1	0	0	1	1	0	1
autokeras	1	0	0	0	1	0	0	0	1	0	1	0	0	0	0	0	0	0	1	1	0	1
auto-sklearn	1	0	0	0	1	0	0	0	1	0	1	0	0	0	0	0	0	0	1	1	0	1
bagging	1	0	0	0	0	1	1	0	0	0	1	0	0	0	0	0	1	0	0	0	1	1
bert	2	1	1	0	0	1	1	1	0	0	1	0	0	1	0	0	0	2	0	1	1	2
bi-bert	1	0	0	0	0	1	1	0	0	0	1	0	0	0	0	0	0	1	0	0	1	1
bi-lstm	3	1	2	0	0	1	1	2	0	0	2	0	0	1	0	0	0	0	3	2	1	3
bim	3	1	0	2	0	1	1	1	1	0	2	0	0	0	0	1	0	0	3	1	1	3
bnn	1	1	0	0	0	1	1	0	0	1	0	0	0	0	0	0	1	0	0	0	0	1
bns	1	1	0	0	0	1	0	1	0	0	1	0	0	0	0	0	1	0	0	1	0	1
bpnn	3	3	0	0	0	3	2	0	1	0	2	0	1	0	0	0	3	0	0	2	0	3
catboost-mo.	1	1	1	0	0	0	0	0	1	0	1	0	0	0	0	0	1	0	0	1	0	1
c-bilstm	1	1	1	0	0	0	0	1	0	0	0	0	0	1	0	0	0	0	1	1	0	1
cbow	1	0	0	0	0	1	0	1	0	0	1	0	0	0	0	0	0	1	0	1	0	1
chi-square	1	0	0	0	0	1	0	1	0	0	1	0	0	0	0	0	0	1	0	1	0	1
clustering	1	0	0	0	0	1	0	1	0	0	1	0	0	0	0	0	0	1	0	1	0	1
cnn	4	1	1	0	1	2	3	1	0	0	3	0	0	1	0	0	0	0	4	1	0	4
cpbt	1	1	0	1	0	0	0	1	0	0	1	0	0	0	0	0	0	0	1	1	0	1
cramer’s v	2	0	0	0	1	1	0	1	1	0	2	0	0	0	0	0	0	2	0	1	0	2
cv	2	2	0	0	2	0	0	0	2	0	0	0	0	0	2	0	2	0	0	1	0	2
deepar	1	1	0	0	0	1	1	0	0	0	1	0	0	0	0	0	1	0	0	1	0	1
dl	1	0	1	0	0	0	0	0	1	0	0	0	0	0	1	0	0	0	1	1	0	1
dnn	2	1	0	0	0	2	1	1	0	1	1	0	1	1	0	0	2	0	0	1	1	2
dt	20	3	4	1	3	13	9	8	4	1	1	13	0	1	4	0	20	0	0	2	0	20
ebt	1	0	0	0	0	1	0	1	0	0	1	0	0	0	0	0	1	0	0	1	0	1
ensemble	3	2	1	0	0	2	0	2	1	1	1	0	0	1	0	0	3	0	0	12	2	3
epsram	1	0	0	1	0	0	0	0	1	0	0	0	1	0	0	0	1	0	0	2	1	1
faxtext	1	0	0	0	0	1	1	0	0	0	1	0	0	0	0	0	0	0	1	1	0	1
ffn	1	0	0	0	0	1	0	0	1	0	1	0	0	0	0	0	1	0	0	0	1	1
flac3d	1	1	0	0	0	1	1	0	0	0	0	0	0	0	0	1	0	0	1	1	0	1
fuzzy appr.	1	1	0	1	0	0	0	0	1	0	1	0	0	0	0	0	0	1	0	0	0	1
gbdt	3	1	0	0	1	2	1	1	1	0	3	0	0	0	0	0	2	1	0	1	0	3
gcn	1	1	0	0	0	1	0	0	1	0	1	0	0	0	0	0	0	0	1	1	1	1
glove	1	0	0	0	0	1	1	0	0	0	1	0	0	0	0	0	0	1	0	1	0	1
gru	1	1	1	0	0	0	0	1	0	0	1	0	0	0	0	0	1	0	0	0	1	1
hcpc	1	1	0	0	0	1	0	1	0	0	1	0	0	0	0	0	0	1	0	1	0	1
ica	1	0	0	0	0	1	1	0	0	0	1	0	0	0	0	0	0	1	0	1	0	1
it2f–ahp	1	1	0	1	0	0	0	1	0	0	0	0	0	0	0	1	0	1	0	0	0	1
it2fd	1	0	0	1	0	0	0	1	0	0	0	0	0	0	0	1	0	1	0	1	0	1
k-means	1	1	0	0	0	1	0	1	0	0	1	0	0	0	0	0	0	1	0	1	0	1
knn	13	1	3	0	2	8	5	6	2	0	8	0	0	2	3	0	13	0	0	1	0	13
ksvm	1	1	0	0	0	1	1	0	0	0	1	0	0	0	0	0	1	0	0	8	1	1
lcca	1	0	0	0	0	1	0	1	0	0	1	0	0	0	0	0	0	1	0	0	0	1
lin_r	6	1	1	1	0	4	1	3	2	0	4	0	1	0	1	0	6	0	0	1	0	6
log_r	7	1	4	0	1	3	1	5	2	0	6	0	0	2	0	0	7	0	0	1	0	7
lstm	4	1	3	0	0	1	0	4	0	0	1	0	0	3	0	0	0	0	4	4	0	4
mlaeld	1	0	0	0	0	1	1	0	0	0	0	0	0	0	1	0	0	0	1	6	1	1
mlp	1	0	0	0	0	1	1	0	0	0	1	0	0	0	0	0	1	0	0	4	0	1
montecarlo	1	1	0	0	0	1	1	0	0	0	1	0	0	0	0	0	0	1	0	1	0	1
mcda-c	1	1	0	1	0	0	0	0	1	0	1	0	0	0	0	0	0	1	0	0	0	1
mosma	1	0	0	0	0	1	0	1	0	0	1	0	0	0	0	0	1	0	0	0	1	1
nb	3	1	0	0	1	2	1	1	1	1	0	2	0	0	0	0	3	0	0	1	0	3
nbc	6	0	3	0	0	3	3	3	0	0	4	0	0	2	0	0	6	0	0	2	0	6
nd	2	2	0	1	1	0	1	0	1	0	2	0	0	0	0	0	0	0	2	4	1	2
nlp	4	1	2	0	0	2	0	4	0	0	2	0	0	2	0	0	0	4	0	1	0	4
nltk	1	0	1	0	0	0	0	1	0	0	0	0	0	1	0	0	0	1	0	4	0	1
one hot enc.	2	0	1	0	0	1	0	1	1	0	2	0	0	0	0	0	0	2	0	1	0	2
pca	5	2	0	0	0	5	2	3	0	1	1	4	1	0	0	0	0	5	0	3	0	5
pca-ahp	1	1	0	0	0	1	1	0	0	0	0	0	1	0	0	0	0	1	0	3	0	1
pls-sem	1	1	0	0	1	0	0	0	1	0	1	0	0	0	0	0	0	1	0	1	0	1
rf	15	9	4	0	2	9	5	6	4	0	10	0	0	1	4	0	15	0	0	0	1	15
rl	1	1	0	1	0	0	1	0	0	0	1	0	0	0	0	0	0	0	1	9	2	1
ros	1	0	0	0	0	1	0	1	0	0	1	0	0	0	0	0	0	0	1	0	0	1
sae	1	0	0	0	0	1	1	0	0	0	1	0	0	0	0	0	0	1	0	1	0	1
satellite-based meas.	1	1	0	0	0	1	1	0	0	0	1	0	0	0	0	0	0	0	1	0	0	1
scibert	1	0	0	0	0	1	1	0	0	0	1	0	0	0	0	0	0	1	0	0	0	1
scikit-learn	1	0	1	0	0	0	0	1	0	0	1	0	0	0	0	0	0	0	1	0	1	1
sd	1	1	0	0	0	1	1	0	0	1	0	0	0	0	0	0	0	1	0	1	0	1
swpl	1	1	1	0	0	0	0	0	1	0	0	0	0	0	1	0	0	0	1	0	0	1
smote	1	0	1	0	0	0	0	0	1	0	1	0	0	0	0	0	0	0	1	1	0	1
sqp	1	0	1	0	0	0	0	1	0	0	0	0	0	1	0	0	0	1	0	1	0	1
srvm	1	1	0	0	0	1	1	0	0	1	0	0	0	0	0	0	1	0	0	0	0	1
svm	15	2	4	0	2	8	3	8	3	0	11	0	0	2	1	0	15	0	0	10	2	15
svr	1	0	0	0	0	1	0	1	0	0	0	0	0	1	0	0	1	0	0	1	0	1
tf-idf	3	0	1	0	0	2	1	2	0	0	2	0	0	1	0	0	0	3	0	1	0	3
tokenitation	1	0	1	0	0	0	0	1	0	0	1	0	0	0	0	0	0	1	0	3	1	1
topsis	1	0	0	0	0	1	1	0	0	0	1	0	0	0	0	0	0	1	0	1	0	1
3d motion	1	1	0	0	1	0	0	1	0	0	0	0	0	0	1	0	0	0	1	1	0	1
vr	3	0	1	1	0	0	0	0	0	0	2	0	0	0	0	2	0	2	1	0	1	3
wbs-rbs	1	1	1	0	0	0	0	0	1	0	0	0	1	0	0	0	0	1	0	1	0	1
word2vec	4	2	1	2	0	0	0	2	0	1	0	0	0	1	0	0	1	3	0	1	0	4
yolo-v5	2	0	0	0	1	0	0	1	0	0	0	0	0	0	0	0	0	1	1	2	1	2

## Appendix E

**Table A5:** Correlation matrix (*l_ij_*) and analysis of significance. The bold numbers express the significance of the correlation with p-values < 0.05). x1–x4 Study objective (classifying, decision making, monitoring, and predicting); x5–x7 Field (construction processes, occupational accidents, and risk management); x8–x14 Data type (project, institutional data, interview, literature, text, signal and video, and simulation); x15–x17 DM type (supervised, unsupervised, and other); x18–x20; Resource type (construction and H&S processes, environment, plant, and machinery). Source: Authors’ processing by Scopus and ProQuest archives and R.

Variable	x1	x2	x3	x4	x5	x6	x7	x8	x9	x10	x11	x12	x13	x14	x15	x16	x17	x18	x19	x20
x1	1.0	−0.1	**0.5**	**0.7**	**0.6**	**0.9**	**0.6**	−0.1	**0.4**	**0.8**	−0.1	**0.8**	**0.6**	−0.1	**0.8**	0.0	0.1	0.2	0.1	0.0
x2	−0.1	1.0	−0.1	−0.2	−0.2	−0.1	0.1	0.1	−0.1	−0.1	0.2	−0.1	−0.1	**0.5**	−0.1	0.1	0.2	0.0	0.1	0.2
x3	**0.5**	−0.1	**1.0**	**0.6**	**0.6**	**0.6**	**0.7**	−0.1	**0.5**	**0.7**	**−0.1**	**0.3**	**0.7**	−0.1	**0.7**	−0.2	0.0	0.1	0.1	−0.1
x4	**0.7**	−0.2	**0.6**	**1.0**	**0.9**	**0.8**	**0.6**	0.0	**0.6**	**0.9**	**0.0**	**0.4**	**0.8**	−0.1	**0.9**	−0.1	−0.2	0.1	0.1	0.0
x5	**0.6**	−0.2	**0.6**	**0.9**	**1.0**	**0.6**	**0.5**	0.0	**0.6**	**0.8**	**0.0**	**0.3**	**0.8**	−0.1	**0.8**	−0.2	0.0	0.1	0.1	0.0
x6	**0.9**	−0.1	**0.6**	**0.8**	**0.6**	**1.0**	**0.6**	−0.1	**0.5**	**0.9**	**−0.1**	**0.7**	**0.6**	−0.1	**0.8**	0.1	0.0	**0.2**	0.1	0.0
x7	**0.6**	0.1	**0.7**	**0.6**	**0.5**	**0.6**	**1.0**	−0.1	**0.3**	**0.7**	**0.2**	**0.2**	**0.7**	−0.1	**0.8**	**−0.2**	−0.1	0.1	0.2	0.0
x8	**−0.1**	0.1	**−0.1**	**0.0**	**0.0**	**−0.1**	**−0.1**	1.0	**0.0**	**−0.2**	**−0.1**	**0.0**	**−0.1**	−0.1	0.0	0.0	−0.1	0.1	0.0	0.2
x9	**0.4**	−0.1	**0.5**	**0.6**	**0.6**	**0.5**	**0.3**	0.0	**1.0**	**0.5**	**0.0**	**0.1**	**0.6**	0.0	**0.6**	−0.1	−0.1	0.0	−0.1	−0.1
x10	**0.8**	−0.1	**0.7**	**0.9**	**0.8**	**0.9**	**0.7**	−0.2	**0.5**	**1.0**	**−0.1**	**0.5**	**0.7**	−0.1	**0.9**	−0.1	−0.1	0.1	0.2	0.0
x11	**−0.1**	0.2	**−0.1**	**0.0**	**0.0**	**−0.1**	**0.2**	−0.1	**0.0**	**−0.1**	**1.0**	**−0.1**	**0.0**	−0.1	0.0	−0.1	−0.1	0.0	−0.1	0.0
x12	**0.8**	−0.1	**0.3**	**0.4**	**0.3**	**0.7**	**0.2**	0.0	0.1	**0.5**	−0.1	1.0	**0.3**	−0.1	**0.5**	0.1	**0.2**	**0.3**	0.1	0.0
x13	**0.6**	−0.1	**0.7**	**0.8**	**0.8**	**0.6**	**0.7**	−0.1	**0.6**	**0.7**	0.0	**0.3**	1.0	−0.1	**0.8**	−0.2	−0.1	0.0	0.1	0.0
x14	**−0.1**	**0.5**	**−0.1**	**−0.1**	**−0.1**	**−0.1**	**−0.1**	−0.1	**0.0**	**−0.1**	−0.1	**−0.1**	−0.1	1.0	−0.1	0.1	0.2	−0.1	0.1	−0.1
x15	**0.8**	−0.1	**0.7**	**0.9**	**0.8**	**0.8**	**0.8**	0.0	**0.6**	**0.9**	0.0	**0.5**	**0.8**	−0.1	**1.0**	−0.2	−0.2	0.2	0.2	0.1
x16	0.0	0.1	**−0.2**	**−0.1**	**−0.2**	**0.1**	**−0.2**	0.0	−0.1	−0.1	−0.1	0.1	−0.2	0.1	**−0.2**	**1.0**	−0.3	−0.1	−0.2	**−0.2**
x17	0.1	0.2	**0.0**	**−0.2**	**0.0**	**0.0**	**−0.1**	−0.1	−0.1	−0.1	−0.1	**0.2**	−0.1	0.2	−0.2	**−0.3**	1.0	0.1	0.1	0.1
x18	0.2	0.0	**0.1**	**0.1**	**0.1**	**0.2**	**0.1**	0.1	0.0	0.1	0.0	**0.3**	0.0	−0.1	0.2	−0.1	0.1	1.0	**0.5**	**0.6**
x19	0.1	0.1	0.1	0.1	0.1	0.1	0.2	0.0	−0.1	0.2	−0.1	0.1	0.1	0.1	0.2	−0.2	0.1	**0.5**	**1.0**	**0.4**
x20	0.0	0.2	−0.1	0.0	0.0	0.0	0.0	0.2	−0.1	0.0	0.0	0.0	0.0	−0.1	0.1	**−0.2**	0.1	**0.6**	**0.4**	**1.0**

## Appendix F

**Table A6:** Papers by variables. x1–x4 Study objective (classifying, decision making, monitoring, and predicting); x5–x7 Field (construction processes, occupational accidents, risk management); x8–x14 Data type (project, institutional data, interview, literature, text, signal and video, and simulation); x15–x17 DM type (supervised, unsupervised, and other); x18–x20; Resource type (construction and H&S processes, environment, plant, and machinery). Source: Authors’ processing by Scopus and ProQuest archives and R.

Reference	x1	x2	x3	x4	x5	x6	x7	x8	x9	x10	x11	x12	x13	x14	x15	x16	x17	x18	x19	x20
[2]	0	0	0	1	1	0	0	0	0	0	1	0	0	0	0	1	0	1	0	0
[3]	0	0	0	1	0	0	1	0	1	0	0	0	0	0	1	0	1	1	0	0
[4]	0	0	1	0	1	0	0	0	0	1	0	0	0	0	1	0	0	1	0	0
[38]	0	0	0	1	1	0	0	0	1	0	0	0	0	0	2	0	0	1	0	0
[21]	0	0	1	0	1	0	0	0	0	0	0	0	1	0	4	0	0	0	0	1
[41]	1	0	0	0	0	0	1	0	0	0	0	0	1	0	2	0	0	0	0	1
[45]	0	1	0	0	1	0	0	0	0	0	0	0	0	1	0	0	2	0	0	1
[13]	0	0	1	0	0	0	1	0	1	0	0	0	0	0	1	0	0	0	0	1
[44]	0	1	0	0	1	0	0	0	1	0	0	0	0	0	0	0	1	0	0	1
[50]	0	0	0	1	0	1	0	0	1	0	0	0	0	0	0	1	0	0	0	1
[24]	0	0	1	0	0	1	0	0	1	0	0	0	0	0	0	0	1	1	0	0
[55]	0	0	0	1	0	0	1	1	0	0	0	0	0	0	1	0	0	0	1	0
[51]	0	0	0	1	1	0	0	0	1	0	0	0	0	0	6	6	2	0	1	0
[56]	0	0	0	1	1	0	0	1	0	0	0	0	0	0	0	0	1	1	0	0
[58]	0	0	0	1	1	0	0	0	0	0	0	0	0	1	0	0	1	0	0	1
[42]	0	0	0	1	1	0	0	0	1	0	0	0	0	0	2	0	1	0	0	1
[37]	0	0	1	0	1	0	0	0	1	0	0	0	0	0	0	0	1	0	0	1
[19]	0	0	0	1	1	0	0	0	1	0	0	0	0	0	1	0	0	0	0	1
[26]	1	0	0	0	0	1	0	0	1	0	0	0	0	0	0	2	0	1	0	0
[70]	0	0	0	1	1	0	0	0	1	0	0	0	0	0	0	0	1	0	0	1
[27]	0	0	0	1	1	0	0	0	1	0	0	0	0	0	1	3	0	0	0	1
[71]	0	0	0	1	1	0	0	0	0	0	0	0	1	0	4	0	1	0	0	1
[43]	0	0	0	1	1	0	1	0	0	0	0	0	1	0	0	1	0	0	0	1
[15]	0	0	0	1	1	0	0	0	1	0	0	0	0	0	4	1	1	0	0	1
[59]	0	0	0	1	0	1	0	0	1	0	0	0	0	0	3	2	1	1	0	0
[12]	1	0	0	0	0	1	0	0	1	0	0	0	0	0	6	1	1	1	0	0
[52]	0	0	0	1	0	1	0	0	0	0	0	1	0	0	4	0	1	1	0	0
[14]	0	1	0	0	1	0	0	0	1	0	0	0	0	0	0	0	1	1	0	0
[46]	0	0	0	1	0	1	0	0	1	0	0	0	0	0	6	0	0	1	0	0
[16]	0	0	0	1	0	1	0	0	1	0	0	0	0	0	3	5	0	1	0	0
[40]	1	0	0	0	0	1	0	0	1	0	0	0	0	0	1	0	0	1	0	0
[28]	0	0	0	1	0	1	0	0	1	0	0	0	0	0	7	0	0	1	0	0
[22]	1	0	0	0	0	1	0	0	0	0	0	1	0	0	0	1	0	1	0	0
[23]	0	0	0	1	0	1	0	0	1	0	0	0	0	0	0	4	1	1	0	0
[29]	0	0	1	0	0	0	1	0	1	0	0	0	0	0	6	0	2	1	0	0
[25]	1	0	0	0	0	1	0	0	0	0	0	1	0	0	6	4	0	1	0	0
[17]	1	0	0	0	0	1	0	0	0	0	0	1	0	0	3	2	5	1	0	0
[47]	0	0	0	1	0	1	0	0	1	0	0	0	0	0	4	4	0	1	0	0
[20]	1	0	0	0	0	1	0	0	1	0	0	0	0	0	1	0	2	1	0	0
[30]	1	0	0	0	1	0	0	0	0	0	0	0	1	0	3	0	0	1	0	0
[64]	0	0	1	0	0	0	1	0	1	0	0	0	0	0	0	0	1	1	0	0
[31]	0	0	0	1	0	1	0	0	1	0	0	0	0	0	1	0	0	1	0	0
[32]	0	0	1	0	0	0	1	0	1	0	0	0	0	0	1	0	0	1	0	0
[57]	0	1	0	0	0	0	1	0	1	0	0	0	0	0	0	1	0	1	0	0
[48]	0	1	0	0	0	0	1	0	0	0	1	0	0	0	3	0	0	1	0	0
[65]	0	1	0	0	0	0	1	0	1	0	0	0	0	0	0	1	0	1	0	0
[60]	0	0	0	1	1	0	0	1	0	0	0	0	0	0	2	1	0	0	0	1
[68]	0	0	0	1	1	0	0	0	0	0	1	0	0	0	1	1	0	1	0	0
[33]	0	0	1	0	0	0	1	0	1	0	0	0	0	0	0	1	0	0	1	0
[18]	0	0	0	1	1	0	0	0	1	0	0	0	0	0	8	1	0	0	1	0
[63]	0	0	0	1	1	0	0	0	1	0	0	0	0	0	1	0	0	0	1	0
[49]	0	1	0	0	0	1	0	0	1	0	0	0	0	0	0	0	1	1	0	0
[34]	0	1	0	0	0	1	0	0	0	0	0	0	0	1	0	2	2	1	0	0
[62]	0	0	0	1	0	0	1	0	1	0	0	0	0	0	1	0	0	1	0	0
[35]	0	0	0	1	0	0	1	0	1	0	0	0	0	0	4	0	0	1	0	0
[36]	1	0	0	0	0	0	1	0	1	0	0	0	0	0	4	1	1	1	0	0
[39]	1	0	0	0	0	0	1	0	0	0	0	0	1	0	0	0	2	1	0	0
[69]	0	0	0	1	0	1	0	0	1	0	0	0	0	0	1	0	0	1	0	0
[66]	0	0	1	0	0	0	1	0	1	0	0	0	0	0	0	1	0	0	1	0
[67]	0	1	0	0	0	0	1	0	1	0	0	0	0	0	0	1	1	0	1	0
[61]	0	0	0	1	0	1	0	0	1	0	0	0	0	0	7	0	0	0	0	1
[53]	0	1	0	0	1	1	0	1	0	0	0	0	0	0	1	0	0	1	0	0
[54]	0	0	0	1	1	0	0	0	1	0	1	1	0	0	0	1	1	0	1	0
